# Intrathecal administration of TRPA1 antagonists attenuate cyclophosphamide-induced cystitis in rats with hyper-reflexia micturition

**DOI:** 10.1186/s12894-016-0150-x

**Published:** 2016-06-17

**Authors:** Zhipeng Chen, Shuqi Du, Chuize Kong, Zhe Zhang, Al-dhabi Mokhtar

**Affiliations:** China Medical University, No. 77 Puhe Road, Shenyang North New Area 110122, Shenyang, Liaoning Province People’s Republic of China; Department of Urology, The First Affiliated Hospital of China Medical University, No. 155 Nanjing North Street, Heping District 110001, Shenyang, Liaoning Province People’s Republic of China

**Keywords:** TRPA1, Antagonist, Urinary bladder, Cystitis, Rats

## Abstract

**Background:**

The activation of TRPA1 channel is implicated in hyper-reflexic micturition similar to overactive bladder. In this study, we aimed to investigate the effects of blocking TRPA1 via intrathecal administration of antagonists on the afferent pathways of micturition in rats with cystitis.

**Methods:**

The cystitis was induced by intraperitoneal cyclophosphamide administration. Cystometry was performed in control and cystitis rats, following the intrathecal injection of the TRPA1 antagonists HC-030031 and A-967079. Real-time PCR, agarose gel electrophoresis, western blotting and immunohistochemistry were used to investigate the levels of TRPA1 mRNA or protein in the bladder mucosa and L6-S1 dorsal root ganglia (DRG).

**Results:**

Edema, submucosal hemorrhaging, stiffness and adhesion were noted during removal of the inflamed bladder. The expression of TRPA1 mRNA and protein was higher in the cystitis group in both the mucosa and DRG, but the difference was significant in the DRG (*P* < 0.05). Intrathecal administration of HC-030031 and A-967079 decreased the micturition reflex in the cystitis group. A 50 μg dose of HC-030031 increased the intercontraction interval (ICI) to 183 % of the no-treatment value (*P* < 0.05) and decreased the non-voiding contraction (N-VC) to 60 % of control (*P* < 0.01). Similarly, the treatment with 3 μg A-967079 increased the ICI to 142 % of the control value (*P* < 0.05) and decreased the N-VC to 77 % of control (*P* < 0.05). The effects of both antagonists weakened approximately 2 h after injection.

**Conclusions:**

The TRPA1 had a pronounced upregulation in DRG but more slight in mucosa in rat cystitis. The blockade of neuronal activation of TRPA1 by intrathecal administration of antagonists could decrease afferent nerve activities and attenuated detrusor overactivity induced by inflammation.

## Background

The transient receptor potential (TRP) channel A1 is a non-selective ion channel that can cause an influx of cations into the cell when activated. It is localized predominantly in small-diameter primary sensory neurons of the dorsal root ganglion and trigeminal ganglion [1–3]. The TRPA1 receptor has been shown to play crucial roles in sensory conducting mechanisms in the neural, respiratory, digestive and other systems as a possible mechanosensitive receptor, nociceptor or cold receptor [4–6]. Based on previous studies, TRPA1 has been described as an essential gatekeeper, transducer and amplifier of inflammation and pain [7, 8].

The main syndrome of acute cystitis is urinary frequency, urgency and dysuria in addition to the impairment of patient quality of life. Chemical cystitis is the key adverse effect observed with cyclophosphamide (CY) chemotherapy, and it results from the formation of acrolein, which is a known agonist of TRPA1 [9, 10]. The TRPA1 channel has been suggested to mediate mechanical and nociceptive sensitivity in both physiological and pathological states of the lower urinary tract [11]. In previous studies, we found that intravesical injection of TRPA1 agonists induced hyper-reflexic micturition similar to overactive bladder [12]. Alterations of the TRPA1 channel are known to contribute to mechanical hypersensitivity in primary sensory nerve endings [13]. It is still debated whether the TRPA1 located in neurons become sensitized to nociceptive or mechanical responses in response to visceral inflammation. We hypothesize that the TRPA1 in primary sensory neurons functions as a mechanical or nociceptive receptor and its activation may enhance afferent nerve activities induced by overactive bladder. Therefore the blockade of the TRPA1 channel may be a potential therapeutic target for bladder overactivity.

Thus the present research was conducted to establish the animal model of acute cystitis to assess alterations in the expression and function of TRPA1. We injected intrathecally the highly specific TRPA1 antagonists HC-030031 and A-967079 to evaluate the involvement of TRPA1 in pathological micturition reflex. Two issues were addressed: First, most antagonists have been administered via intravenous or intragastric routes, while the use of intrathecal administration has been rarely reported. The local intrathecal administration could reduce severe gastrointestinal and cardiovascular adverse effects, thus facilitating the identification of potential therapeutic strategies; second, if TRPA1 is involved in the pathological micturition reflex, novel therapeutic drugs could be developed to target this protein.

## Methods

### Animals and ethics statement

Female Sprague–Dawley rats (weight 210 to 245 g) were used. The production, feeding and nursing of the rats were performed by Experimental Animal Center of China Medical University (Certification No.2013002R) and the study was specifically approved by the Animal Ethics Committee of China Medical University. All surgeries were performed under anesthesia, and all efforts were made to minimize suffering. The animals were killed under anesthesia (60 mg/kg sodium pentobarbital) following the recommendations of the US National Institutes of Health.

These rats were housed in standard polypropylene cages, with four animals per cage, at a temperature-controlled, humidity-controlled room and 12–12 light/dark cycle. Cystitis was induced via an intraperitoneal injection of 300 mg/kg CY (Hengrui, China). Sham-treated rats received normal saline (Huaren, China). The expression and function studies were performed 48 h after the injection of CY. For cystometry, the rats were anesthetized via a subcutaneous injection of 1.2 g/kg urethane (Sigma, USA).

### Histopathology

The excised bladder was fixed immediately in 4 % buffered formaldehyde for approximately 24 h, dehydrated in a series of alcohol concentrations, cleared in xylene, embedded in paraffin blocks (Thermo excelsior ES, USA), serially sectioned to a thickness of 5 μm and placed on coated slides. Subsequently, the tissue sections were stained with hematoxylin and eosin (H&E) dehydrated in a graded ethanol series, cleared in xylene, and coverslipped using mounting medium. The slides were examined by light microscopy (Olympus IX71, Japan).

### Quantitative Reverse Transcriptase-Polymerase Chain Reaction and AGE (Agarose Gel Electrophoresis)

The L6-S1 DRG and urinary bladder were harvested (*n* = 6). Under a stereoscopic microscope, the bladder mucosa was separated from the muscular layer. Total RNA was extracted using an RNeasy mini kit and RNase-free DNase kit (Qiagen, Germany). The detail steps were described as before [12]. The 2 % agarose gel (Invitrogen, USA) was resolved in 1 × TBE buffer (Tris, Boric acid, EDTA, pH 7.5). The PCR products supplemented with loading buffer were electrophoresed in a horizontal apparatus (Bio-Rad, USA) at 150 V for 30 min. The bands were imaged using InGenius Imager (Syngene, USA) under UV light.

### Detection of TRPA1 expression by western blotting analysis and localization by immunohistochemistry

The DRG and bladder mucosa were dissolved in RIPA Lysis Buffer (Beyotime, Shanghai, China) containing protease inhibitor (*n* = 4). The protein homogenate was centrifuged at 4 °C and 12,000 rpm for 30 min. The Bio-Rad DC protein assay (model 680; Bio-Rad) was used to detect the concentration via a BSA standard. Equal proteins were separated by 8 % SDS-PAGE and then transferred onto a PVDF membrane. Primary antibodies were incubated on the membranes for TRPA1 (Abcam, ab68848) (1:400) and GAPDH (Santa Cruz) (1:1,000) overnight at 4 °C in TBST and secondary antibodies were incubated at 37 °C for 2 h. The proteins were detected in an ECL detection system (UVP Inc., Cambridge, UK) through enhanced chemiluminescence detection reagents. We used EC3 Imaging System (UVP Inc.) to catch up the specific bands, and relative intensities of all bands were quantified using Image J software. The ratio between the optical density of the TRPA1 and GAPDH protein was calculated as relative content and expressed graphically.

The slides were treated with 3 % hydrogen peroxide to block endogenous peroxidase and with Protein Block Serum-Free to block nonspecific protein binding. The TRPA1 antibody (Abcam, ab68848) was used as a primary antibody at a dilution of 1:300 for 16 h at 4 °C. After washing, the slides were incubated with the horseradish peroxidase (HRP) conjugated secondary antibody (Maixin, Fuzhou, China) for 30 min at room temperature. Then, DAB was applied for color development. With the use of the Image-Pro plus freeware, the intensity of staining was quantitatively determined in selected areas on digital image of each slice by normalizing with background value.

### Cystometry

Prior to the study, all of the rats had free access to food and water. The rats were fixed to the plate in the prone position. We separated the muscle around the fourth lumbar (L4) spinous process, removed the spinous process and adjacent vertebra, exposed the clearance between L3 and L4 and punctured the yellow ligament gently with a microneedle. Next, a PE-10 catheter’s tip was handled by fire in order to avoid neuronal damage; we inserted the catheter parallel to the longitudinal axis through the crevasse and fixed it on the neck of the animal. The rats were carefully turned to the supine position, and another PE-50 catheter was inserted into the bladder through the dome. This catheter was connected through a three-way stopcock to a microinjection pump (Beyond, China) and pressure transducer (RM6240, Chengyi, China) as previously described [12]. We examined the effects of the TRPA1 antagonists via an intrathecal injection of 50 μg HC-030031 and 3 μg A-967079 in normal and cystitis rats [14]. The intravesical pressure was amplified (RM6240, Chengyi) and recorded using a computer (ThinkPad, China). Normal saline (37 °C, pH 7.0 to 7.2) was infused continuously into the bladder at 45 μl/min. The following parameters were calculated as the average of five or six stable successive micturition cycles from the normal (*n* = 4) and cystitis groups (*n* = 4): baseline pressure (BT), pressure threshold (PT), compliance, intercontraction interval (ICI), micturition pressure (MP), and non-voiding contraction (N-VC). N-VC were defined as a rhythmic intravesical pressure increase greater than 5 mmHg from baseline pressure without release of saline from the urethra [12].

### Chemicals

The TRPA1 antagonists HC-030031 and A-967079 were obtained from Sigma-Aldrich. Both antagonists were dissolved in 10 % dimethylsulfoxide (DMSO), 5 % Tween 80 and 85 % sterile saline solution. The durgs or vehicle (sterile saline solution) were injected by intrathecal route contained 0.5 % DMSO. The administration of vehicle did not display any effect.

### Statistical analysis

All of the data were presented as the mean ± standard error (SD). The statistical analysis was performed using Student’s *t*-test and one-way analysis of variance, with a significance threshold of *P* < 0.05.

## Results

### Histological analysis

**Fig. 1:**
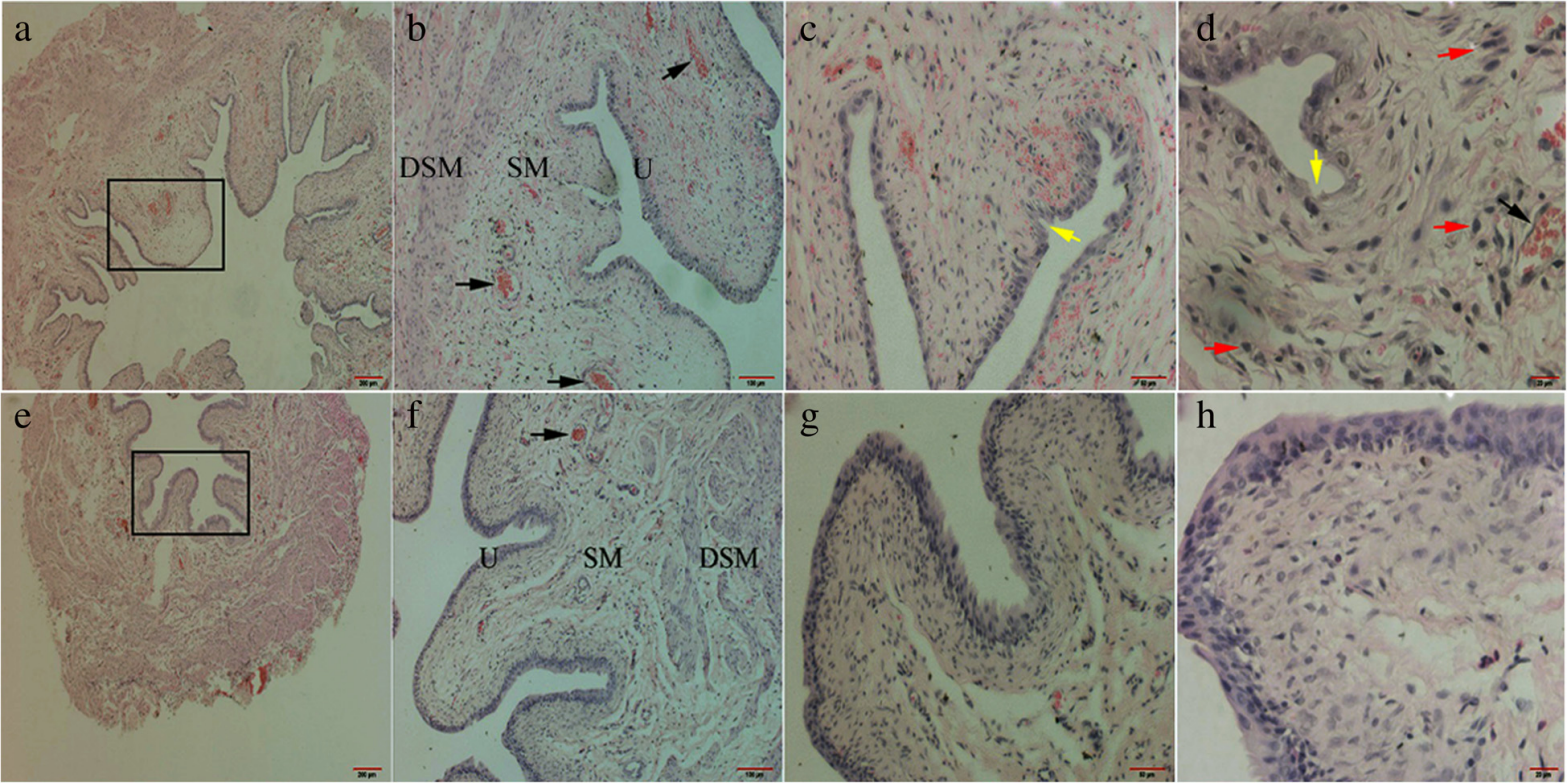
Characteristic histological findings in a cross-section of the bladder wall. “**a** to **d**” show the inflamed bladder mucosa consisting of the urothelium (U), submucosa (SM) and detrusor smooth muscle (DSM). Compared with the controls (**e** to **h**), severe submucosal edema (hollow box), hemorrhagia (black arrow), ulceration (yellow arrow), congestion and inflammatory cell infiltrates (red arrow) were observed in CY-treated rats (**a** to **d**). H&E, reduced from 40×, 100×, 200× to 400×

The model of cystitis was induced with CY, which has been used worldwide [15]. Histopathology was conducted 48 h after intraperitoneal injection of saline and CY (Fig. 1). Macroscopically, the inflamed bladder had a much thicker wall and weighed more compared with the normal bladder. Edema, congestion, stiffness and adhesion were noted during removal of the inflamed bladder. In CY-treated group, a thin epithelium, intense edema (Fig. 1a), congestion (Fig. 1b), submucosal hemorrhaging (Fig. 1b), abrasion (Fig. 1c) were markedly increased in large areas. Moreover, the infiltration of large numbers of mononuclear inflammatory cells in the edematous mucosa suggested that CY induced cystitis (Fig. 1d). However, in the control group, microscopic examination of the bladder revealed the gross and histopathological features of the mucosa, urothelium, submucosa and detrusor smooth muscle (Fig. 1e-h).

### Quantification of TRPA1 mRNA level

**Fig. 2:**
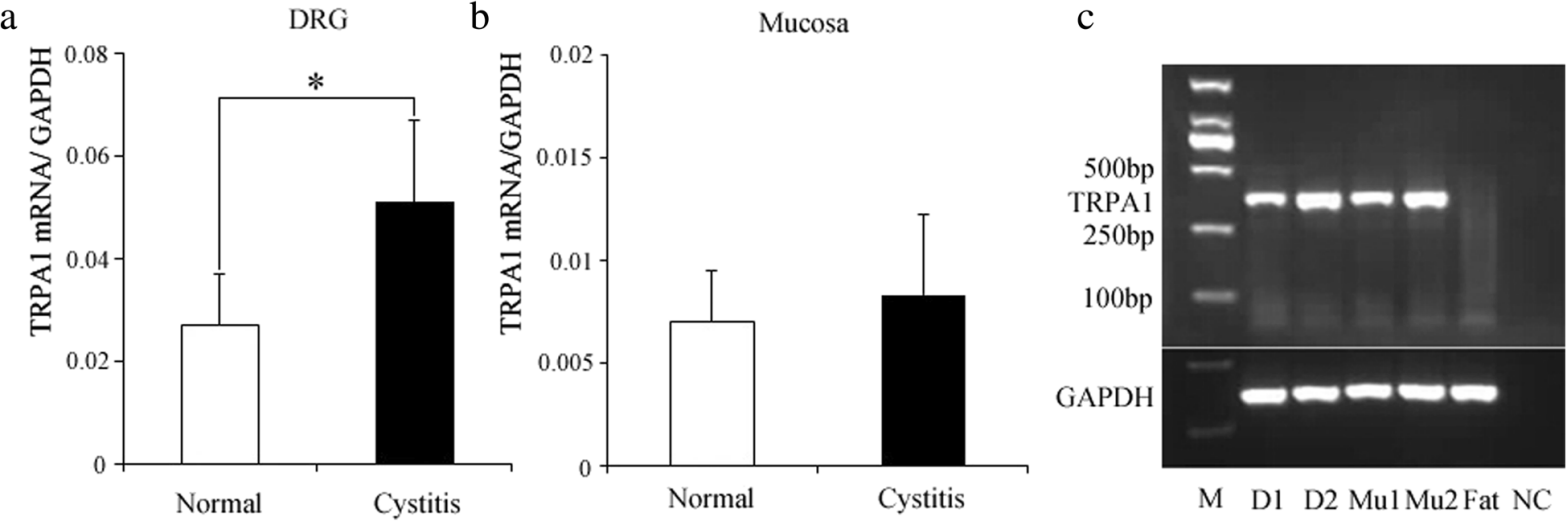
The mRNA expression levels of TRPA1 in DRG and bladder mucosa were determined. The TRPA1 mRNA level in DRG (**a**) and mucosa (**b**) during cystitis (filled column, *n* = 6) was 1.89 times and 1.19 times greater than that in the control group (open column, *n* = 6), respectively. The PCR products of the DRG and mucosa (Mu) showed bands of TRPA1 at 358 bp in electrophoresis (**c**). M: marker; Mu1: normal bladder mucosa; Mu2: inflammatory bladder mucosa; D1: normal dorsal root ganglion; D2: inflammatory dorsal root ganglion; NC: no reverse-transcriptase negative control

The levels of TRPA1 mRNA were quantified using the housekeeping gene GAPDH as an internal standard(TRPA1/GAPDH). The values for the normal and cystitis groups were 0.027 ± 0.01 and 0.051 ± 0.02 in the DRG (Fig. 2a) and 0.007 ± 0.003 and 0.008 ± 0.004 in the mucosa (Fig. 2b), respectively. The expression of TRPA1 mRNA was higher in the cystitis group in both the mucosa and DRG, but the difference was significant in the DRG (*P* = 0.014). Its expression level for DRG/mucosa was 3 ~ 6:1, demonstrating the more abundant expression in the DRG. We also observed that the TRPA1 had much more expression in the cystitis mucosa than the normal via AGE and the fat tissue did not express TRPA1 mRNA, although they expressed GAPDH mRNA (Fig. 2c).

### Quantification of TRPA1 Protein level

**Fig. 3:**
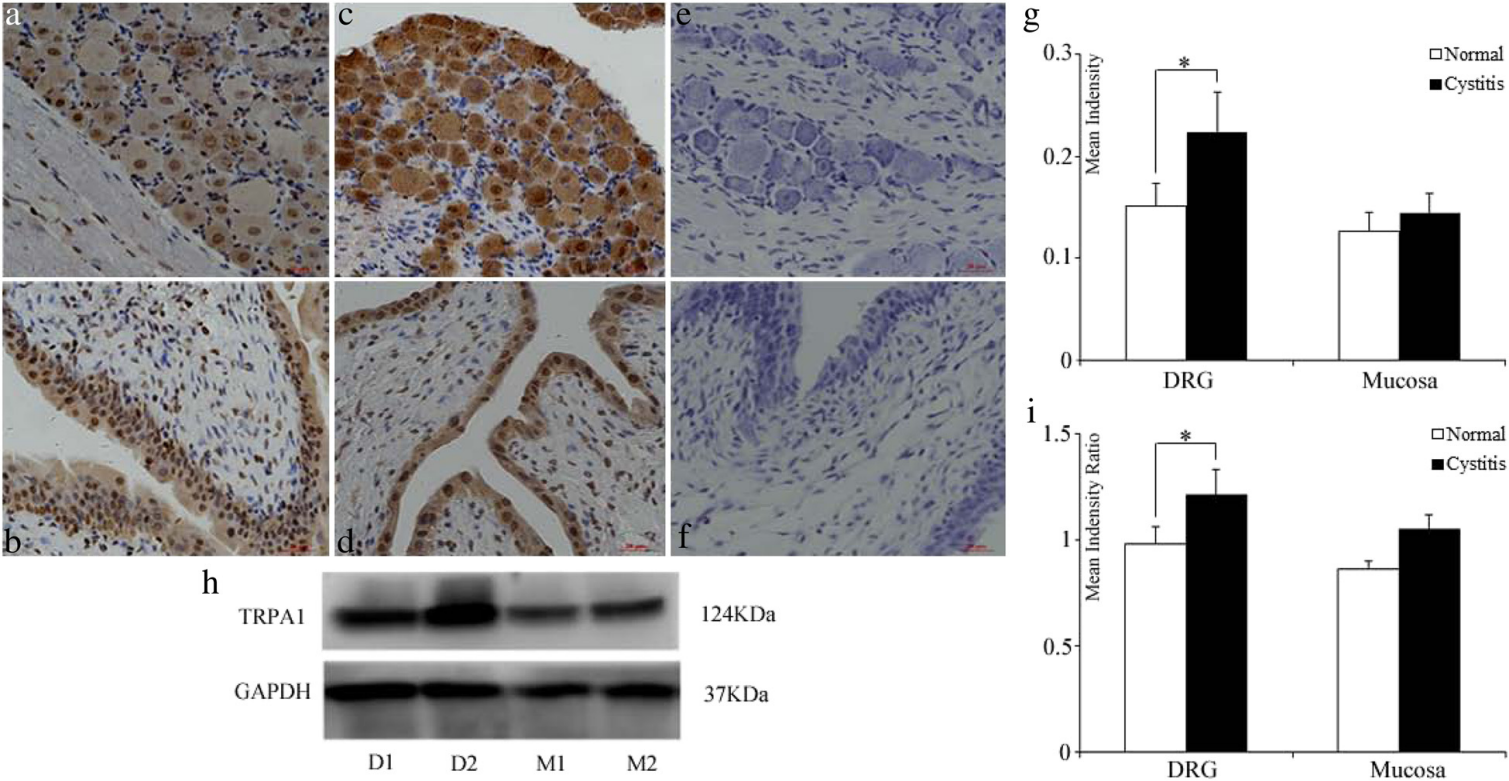
Immunohistochemistry and western blotting analysis of TRPA1 expression in DRG and mucosa. Representative images of TRPA1 immunostaining in DRG (**a**) and mucosa (**b**) of normal groups and cystitis (**c** and **d**, respectively). Negative control of DRG (**e**) and mucosa (**f**). Immunohistochemistry (**g**) and western blotting (**h**, **i**) analysis of TRPA1 expression in DRG and mucosa. Each column represents the mean and vertical lines indicate the SD of 4 animals.*P < 0.05

Immunohistochemistry and western blotting analysis of TRPA1 expression in DRG and mucosa of cystitis and normal group. Representative images of the TRPA1 expression in DRG (Fig. 3a) and mucosa (Fig. 3b) with normal group. DRG (Fig. 3c) and mucosa (Fig. 3d) of the cystitis were also be showed. The immunohistochemistry analysis showed that TRPA1 in DRG was markedly upregulated in the cystitis group (0.224 ± 0.04 vs 0.151 ± 0.02; Fig. 3g; *P* = 0.018) while the TRPA1 protein level in cystitis mucosa did not have any significant alteration (0.145 ± 0.02 vs 0.127 ± 0.02; *P* = 0.4). Accordingly, the western blotting analysis also showed the TRPA1 protein significantly increased in the DRG with cystitis(1.21 ± 0.12 vs 0.98 ± 0.08; Fig. 3h ,i; *P* = 0.018). Similarly to the data observed in the TRPA1 mRNA levels, there was no significant alteration in the protein expression in mucosa(1.05 ± 0.07 vs 0.86 ± 0.04; *P* = 0.14).

### Cystometry of rats with cystitis

**Fig. 4:**
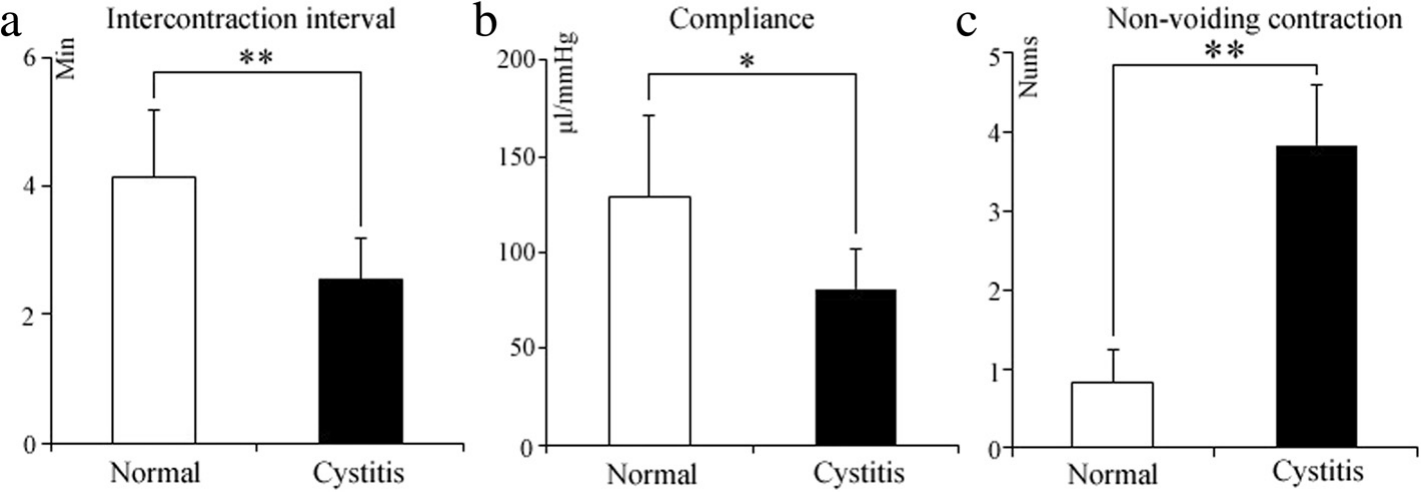
Comparison of experimental results between the cystitis and normal group. The cystitis and normal groups were injected with CY and saline, respectively, and cystometry was performed after 48 h. The values of intercontraction interval (**a**), compliance (**b**) and non-voidingcontraction (**c**) were obtained from the cystitis group and the normal group, respectively; Each column represents the mean and vertical lines indicate the SD of 6 animals. *P < 0.05, ** *P* < 0.01

In the cystometrograms, the ICI of the cystitis group decreased significantly in comparison with the normal group (2.55 ± 0.64 vs 4.16 ± 1.02 min; Fig. 4a; *P* < 0.01). A decrease in bladder compliance with cystitis was observed significantly (80.83 ± 21.42 vs 128.8 ± 42.07 μl/mmHg; Fig. 4b; *P* = 0.03). N-VC increased significantly in the cystitis group compared with the normal group (3.83 ± 0.75 vs 0.833 ± 0.40; Fig. 4c; *P* < 0.01), while the other parameters such as the baseline pressure, pressure threshold and micturition pressure were not significantly different.

### Cystometry of the administered antagonists

**Fig. 5:**
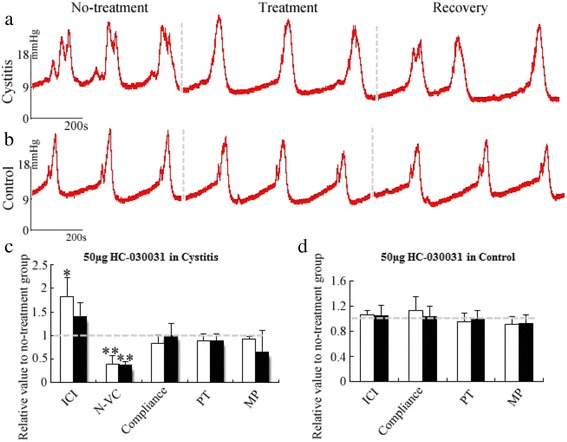
Effects of intrathecal injection of TRPA1 antagonist HC-030031. Representative cystometrograms of intrathecal injection in cystitis (**a**) and control (**b**), respectively. Recovery represents 2 h after intrathecal injection of 50 μg HC-030031. The relative value of 50 μg HC-030031(open column) and the recovery (filled column) compared with no-treatment in cystitis (**c**) and control (**d**) on cystometry parameters, respectively. Each column represents the mean and vertical lines indicate the SD of 4 animals. ICI: intercontraction interval, N-VC: non-voiding contraction, PT: pressure threshold, MP: micturition pressure. *P < 0.05, ** *P* < 0.01

**Fig. 6:**
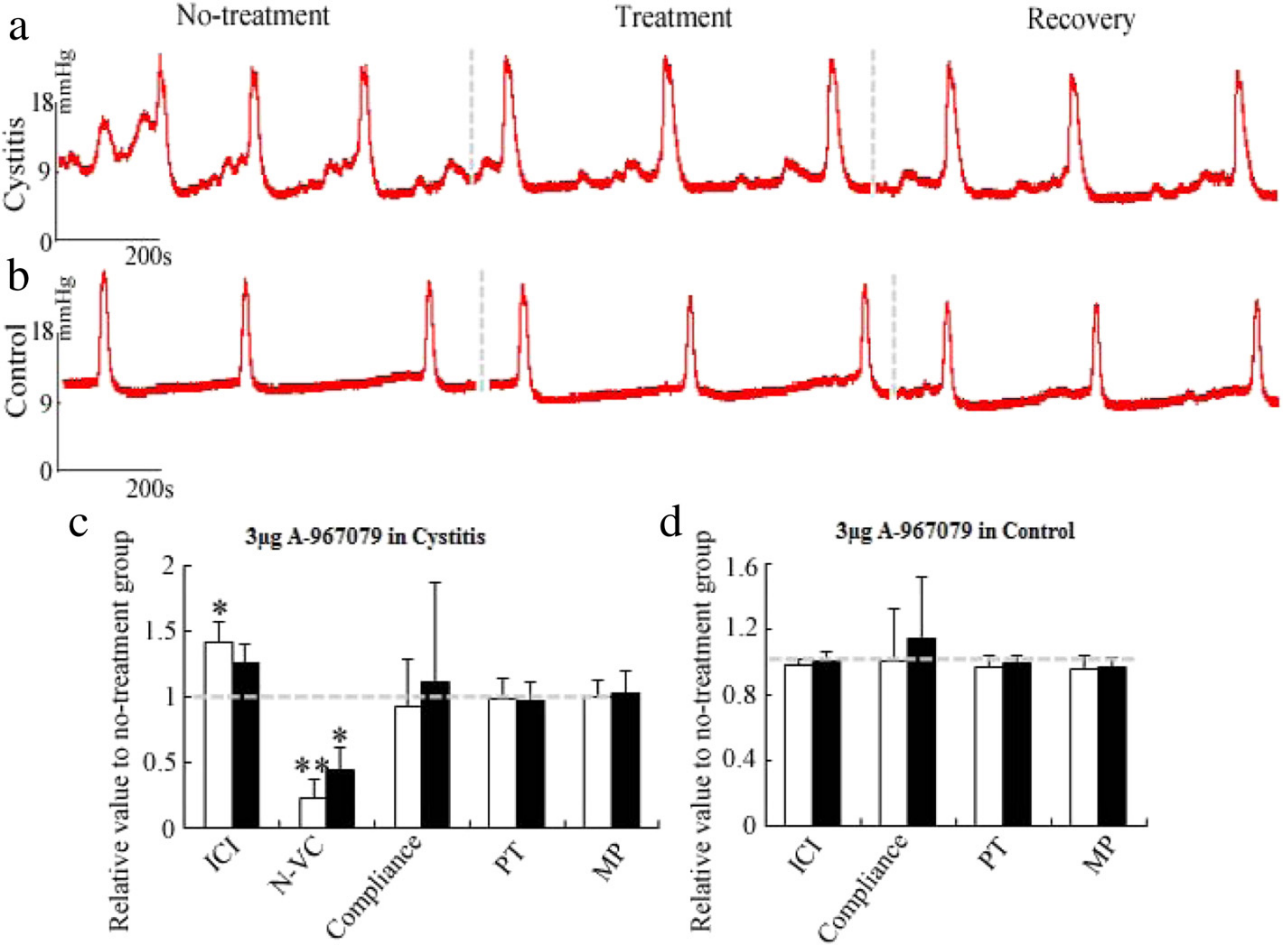
Effects of intrathecal injection of TRPA1 antagonist A967079. Representative cystometrograms of intrathecal injection in cystitis (**a**) and control (**b**), respectively. Recovery represents 2 h after intrathecal injection of 3 μg A967079. The relative value of 3 μg A967079 (open column) and the recovery (filled column) compared with no-treatment in cystitis (**c**) and control (**d**) on cystometry parameters, respectively. Each column represents the mean and vertical lines indicate the SD of 4 animals. ICI: intercontraction interval, N-VC: non-voiding contraction, PT: pressure threshold, MP: micturition pressure. *P < 0.05, ** *P* < 0.01

Figures 5 and 6 showed the changes of cystometry parameters in cystitis and control group by intrathecal administration of 50 μg HC-030031 and 3 μg A-967079, respectively. In the cystitis group (Fig. 5a ,c), a dose of 50 μg HC-030031 increased the ICI to 183 % of the control value (*P* = 0.02). The reduced effectiveness of the drug resulted in a recovery of the ICI to 140 % of its control value after 2 h (*P* = 0.09). The N-VC decreased to 60 % of the control value (*P* < 0.01) after HC-030031 infusion and then increased to 62 % approximately 2 h later (*P* < 0.01). The baseline pressure, pressure threshold, micturition pressure and compliance displayed no significant difference before and after intrathecal injection of the drug. The effects of HC-030031 were apparent approximately 30 min after the intrathecal injection, but the recovery was observed after 2 h.

The intrathecal injection of 3 μg A-967079 in the cystitis (Fig. 6a, c) increased the ICI to 142 % of the control value (*P* = 0.02). After approximately 2 h, the ICI recovered to 126 % of the control value due to the decreased drug strength (*P* = 0.38). N-VC decreased to 77 % of the control (*P* < 0.01) after the infusion of A-967079 and increased to 66 % approximately 2 h later. However, a dose of 50 μg HC-030031 (Fig. 5b, d) and 3 μg A-967079 (Fig. 6b, d) had no obvious effects on the cystometry parameters in the normal group. The effects of antagonists on micturition were presented in Tables 1 and 2.

**Table 1:** The effect of TRPA1 antagonist 50 μg HC-030031 on cystometry parameters of inflammatory rats

						Compliance (μl/mmHg)	N-VC (number)
		BP (mmHg)	PT (mmHg)	MP (mmHg)	ICI (min)		
	No-treatment	13.11 ± 6.14	15.86 ± 6.25	24.33 ± 8.78	3.92 ± 1.41	139.10 ± 49.47	0.25 ± 0.5
Control	Treatment	11.24 ± 3.11	14.25 ± 3.44	21.54 ± 5.34	4.08 ± 1.32	159.15 ± 76.02	0
	Recovery	11.17 ± 3.08	14.86 ± 3.48	21.53 ± 5.14	3.90 ± 0.74	146.46 ± 65.47	0
	No-treatment	10.10 ± 5.74	11.83 ± 6.16	19.11 ± 8.01	2.28 ± 0.51	124.78 ± 49.52	3.25 ± 0.5
Cystitis	Treatment	7.70 ± 3.29	9.83 ± 3.05	17.29 ± 6.87	4.14 ± 1.05*	98.12 ± 25.15	1.25 ± 0.50**
	Recovery	7.39 ± 3.75	10.05 ± 4.25	15.72 ± 7.00	3.10 ± 0.31	136.80 ± 42.92	1.25 ± 0.50**

Results were expressed as mean ± standard error. The “*” or “**” represented the difference was significant between the treatment or recovery and no-treatment in cystometry parameters. *P < 0.05, ** *P* < 0.01

**Table 2:** The effect of TRPA1 antagonist 3 μg A-967079 on cystometry parameters of inflammatory rats

						Compliance (μl/mmHg)	N-VC (number)
		BP (mmHg)	PT (mmHg)	MP (mmHg)	ICI (min)		
	No-treatment	11.83 ± 2.96	12.74 ± 3.4	21.76 ± 1.63	4.80 ± 1.79	91.44 ± 18.19	0.25 ± 0.5
Control	Treatment	11.66 ± 3.32	12.42 ± 3.83	20.82 ± 2.84	4.76 ± 1.96	92.88 ± 43.76	0
	Recovery	11.71 ± 3.19	12.73 ± 3.83	21.19 ± 2.22	4.68 ± 1.68	104.56 ± 40.19	0
	No-treatment	10.54 ± 3.23	11.94 ± 2.88	22.68 ± 4.89	3.64 ± 0.78	80.07 ± 27.31	3.00 ± 0.82
Cystitis	Treatment	10.25 ± 3.99	12.00 ± 4.67	22.68 ± 5.28	5.87 ± 0.93*	67.40 ± 7.66	0.75 ± 0.50**
	Recovery	9.85 ± 4.37	11.46 ± 4.75	21.96 ± 5.75	4.50 ± 1.59	90.86 ± 34.91	1.25 ± 0.50*

Results were expressed as mean ± standard error. The “*” or “**” represented the difference was significant between the treatment or recovery and no-treatment in cystometry parameters. *P < 0.05, ** *P* < 0.01

## Discussion

The present study demonstrates the TRPA1 was expressed in both bladder and DRG (L6-S1) and had a pronounced upregulation in DRG but more slight in mucosa in rat cystitis. The blockade of TRPA1 via intrathecal administration decreased afferent nerve activities and consequently attenuated detrusor overactivity markly. More recently, Tomonori et al. have shown that TRPA1 channel could improve afferent nerve activities of the rat bladder through both Aδ- and C-fibers pathway [16]. TRPA1 channels have been conducted in multiple-sensation modalities at present including mechanical, nociceptive, and thermal sensation in mammal [17–19].

However, the function of TRPA1 as nociceptor in the DRG innervating bladder is really quite controversial and further research is needed. We suppose the activation of TRPA1 receptors in DRG may lead to hyperalgesia, playing a role in enhanced impulse conduction and detrusor overactivity. We observed hematuria, severe submucosal edema, hemorrhage, ulceration, congestion and inflammatory cell infiltration following the intraperitoneal injection of CY for 48 h. The symptoms of overactive bladder, a shortened ICI and an increase in unstable contractions, were observed concomitantly. In the present study, the levels of TRPA1 mRNA and protein in DRG were significantly higher in cystitis group than the control group while the TRPA1 in mucosa were slightly higher than the control group without statistical significance, indicating that the TRPA1 in DRG may play a greater role than in mucosa. Similarly, Andrade also found a higher expression of TRPA1 mRNA in DRG neurons in the study investigating bladder overactivity induced by spinal cord injury [20]. It also be demonstrated that the TRPA1 expression level was significantly higher than bladder mucosa [12]. The possible reason may refer to the TRPA1 in mucosa might not function as nociceptive receptor in the case of cyclophosphamide-induced inflammation. The profound results showed that the modulation of intracellular Ca^2+^ could contribute directly to the elevated gene expression of TRPA1 via an influx through voltage-gated channels and/or endoplasmic reticulum [21, 22]. It has also been found that TRPA1 mediates inflammation, hyperalgesia and visceral hypersensitivity in pancreatitis pain as well as in a model of acute gout [23, 24]. The mechanism could be construed as the TRPA1 in DRG played a crucial role in sensitization of sensory afferent nerves in occurrence of pathological conditions.

Symptoms of overactive bladder such as a shortened ICI and an increase in N-VC were observed in cystitis. We conceive that blocking the TRPA1 in DRG might attenuate the excitability of afferent pathways and thus alleviate detrusor overactivity. Following intrathecal injection of the highly specific TRPA1 antagonists HC-030031 and A-967079,the ICI was extended, and the N-VC was suppressed in cystitis, thereby inhibiting micturition reflex hyperactivity. However, there were no significant changes in BP, PT, MP and compliance in CY-induced rats before and after the application of each TRPA1 antagonists although the ICI was significantly increased and the number of N-VC was significantly decreased. The probable reason is that the cystometry was performed under anesthetized conditions. Interestingly, neither HC-030031 nor A-967079 had a substantial effect on normal urination, suggesting that TRPA1 might not participate in the physiological micturition reflex. More TRPA1 channels might need to be open under pathological conditions, leading to hyperalgesia in the absence of a physiological pain signal. A study by Perin-Martins A demonstrated the mechanisms that contribute to edema and hyperalgesia induced by TRPA1 activation [25]. The antagonists prevent and reverse cystitis, suggesting that TRPA1 is pivotal for the maintenance and development of the inflammatory response and hyperalgesia. The effects of both antagonists persist for approximately two hours, which is consistent with previous findings [26]. When the effects of the antagonists disappeared, ICI returned, indicating that continuous activation of the TRPA1 in DRG neurons is crucial to maintain the nociceptor sensitization elicited by inflammatory stimulation. This proposal is further supported by previous findings showing that TRPA1 mediates sustained hyperalgesic responses in different models [14, 27]. Indeed, it has been demonstrated that spinal blockage of the N-type and P/Q-type VGCC (voltage-gated calcium channel) could attenuate inflammatory and nociceptive events associated with cystitis [10]. Consistent to this notion, TRPA1 located on DRG contributes to the transmission of nociceptive information to second-order neurons in the spinal dorsal horn [14, 26].

Studies have showed that HC-030031 and A-967079 were potentially capable of blocking the effect of TRPA1 with a much higher selectivity than other ion channels [28, 29]. Intrathecal administration of antagonists is rarely reported compared with antisense oligonucleotides when blocking the TRPA1 in rats. We use intrathecal injection, which not only can act directly on DRG but can reduce the side effects associated. However, intrathecal administration is a kind of invasive operation which may also accompany related complications such as infection, so its clinical application might be limited. Nevertheless, the intrathecal administration is also a suitable alternative when faced with refractory bladder diseases. Taken together, our results indicate that TRPA1 especially in DRG plays a key role in the occurrence of cystitis, and therefor intrathecal injection of TRPA1 antagonists might be effective in treating detrusor overactivity. It should be noted that this study has examined only the TRPA1 expression and function in the stage of acute inflammation, while a time-dependent change of TRPA1 after cystitis was not involved. Our data also showed the duration of the drug lasted only two hours, and therefore, further research is needed for possibly clinical applications in future.

## Conclusions

The present findings suggested that TRPA1 might only be involved in pathological rather than physiological micturition reflex. The blockade of neuronal activation of TRPA1 via intrathecal administration could decrease afferent nerve activities and attenuate detrusor overactivity induced by inflammation. Therefore, in multistep sensory pathway, TRPA1 in DRG might be used as a more effective therapeutic target for the treatment of pathological micturition.

## Abbreviations

BP, baseline pressure; CY, cyclophosphamide; DRG, dorsal root ganglia; GAPDH, glyceraldehyde-3-phosphate dehydrogenase; ICI, intercontraction interval; MP, micturition pressure; N-VC, non-voiding contraction; PT, pressure threshold; TRPA1, transient receptor potential channel A1
